# ZBTB33 binds unmethylated regions of the genome associated with actively expressed genes

**DOI:** 10.1186/1756-8935-6-13

**Published:** 2013-05-21

**Authors:** Adam Blattler, Lijing Yao, Yao Wang, Zhenqing Ye, Victor X Jin, Peggy J Farnham

**Affiliations:** 1 Department of Biochemistry & Molecular Biology, Norris Comprehensive Cancer Center, University of Southern California, Los Angeles, CA, 90089, USA; 2 Genetics Graduate Group, University of California-Davis, Davis, CA, 95616, USA; 3 Department of Biomedical Informatics, The Ohio State University, Columbus, OH, 43210, USA

**Keywords:** DNA methylation, Zinc finger proteins, Histone modifications, Transcription factor binding, Epigenetics, Transcriptional regulation

## Abstract

**Background:**

DNA methylation and repressive histone modifications cooperate to silence promoters. One mechanism by which regions of methylated DNA could acquire repressive histone modifications is via methyl DNA-binding transcription factors. The zinc finger protein ZBTB33 (also known as Kaiso) has been shown in vitro to bind preferentially to methylated DNA and to interact with the SMRT/NCoR histone deacetylase complexes. We have performed bioinformatic analyses of Kaiso ChIP-seq and DNA methylation datasets to test a model whereby binding of Kaiso to methylated CpGs leads to loss of acetylated histones at target promoters.

**Results:**

Our results suggest that, contrary to expectations, Kaiso does not bind to methylated DNA in vivo but instead binds to highly active promoters that are marked with high levels of acetylated histones. In addition, our studies suggest that DNA methylation and nucleosome occupancy patterns restrict access of Kaiso to potential binding sites and influence cell type-specific binding.

**Conclusions:**

We propose a new model for the genome-wide binding and function of Kaiso whereby Kaiso binds to unmethylated regulatory regions and contributes to the active state of target promoters.

## Background

Genes are epigenetically regulated by a combination of histone modifications and methylation of CpG dinucleotides near their promoters [1,2]. Promoters that have high levels of DNA methylation always show low activity whereas the relationship of histone methylation and promoter activity differs depending on exactly which residue is methylated. For example, trimethylation of lysine 4 of histone H3 (H3K4me3) correlates with high promoter activity whereas trimethylation of lysine 9 or lysine 27 of histone H3 (H3K9me3 or H3K27me3) correlates with low promoter activity. Recent studies have demonstrated distinct spatial relationships of these two repressive histone modifications with regions of methylated DNA [3,4]. These analyses entailed chromatin immunoprecipitation using antibodies that recognize specific histone modifications followed by bisulfite sequencing of the ChIP DNA (BisChIP-seq). One study showed that H3K27me3 can sometimes be associated with methylated promoters but that the H3K27me3-marked regions are repressed independently of the level of DNA methylation [3]. A similar study by Brinkman et al. [4] found that H3K27me3 and DNA methylation are compatible except at CpG-rich promoters where the two marks are mutually exclusive, supporting a previous study by Komashko et al. [5]. The studies of Komashko et al. also showed a very low overlap of the sets of promoters having high levels of H3K9me3 versus high levels of DNA methylation. From their BisChIP-seq results, Brinkman et al. suggest that H3K9me3 has a high degree of overlap with regions of methylated DNA. However, the majority of H3K9me3 sites in the genome are not at promoter regions and, unfortunately, their analyses did not distinguish the DNA methylation status of promoter versus non-promoter H3K9me3 sites. In summary, although the spatial relationship of these repressive histone methylations has been described, it is not yet clear if DNA methylation and H3K27me3 or H3K9me3 cooperate to repress promoter regions.

**Figure 1:**
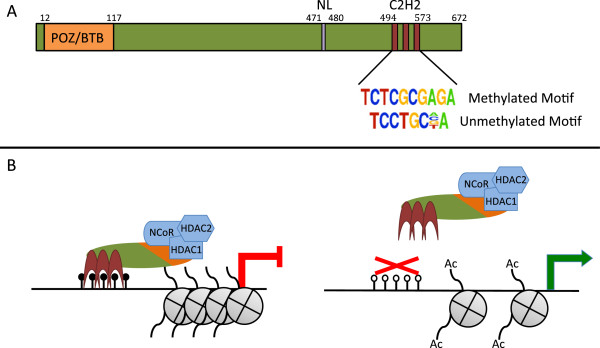
**Structure and function of Kaiso.** (**A**) Kaiso is a 672 amino acid protein, which includes an N-terminal POZ/BTB domain required for protein-protein interactions, a nuclear localization signal, and three tandem C2H2 zinc finger domains responsible for DNA binding; numbers represent the amino acid borders of each domain. Shown below the zinc finger domains are the two motifs to which Kaiso has been shown to bind in vitro. (**B**) The current model for Kaiso’s activity at promoters: in the left figure, Kaiso’s 3 C2H2 zinc finger domains recognize and bind methylated DNA, recruiting the NCoR histone deacetylation complex to the region and causing the loss of active chromatin marks and the repression of the adjacent promoter. The figure on the right shows an unmethylated promoter, which is not recognized by Kaiso. As a result, the NCoR histone deacetylation complex is not recruited to the region and surrounding histones remain acetylated allowing for expression of the promoter.

In addition to repressive histone methylation modifications, active versus inactive promoters can also be distinguished by the acetylation status of histone H3. Active promoters are marked by both acetylated lysine 9 and acetylated lysine 27 on histone H3 (H3K9Ac and H3K27Ac). In fact, H3K9Ac is the modified histone most highly associated with transcriptional activity, as shown in the integrative analysis of a large number of datasets by the ENCODE consortium [6]. Although DNA methylation may not cooperate with repressive histone methylation modifications to silence genes, there may be a functional relationship between DNA methylation and histone deacetylases. For example, it is possible that DNA methylation could be a signal for the binding of a site-specific DNA-binding factor which could in turn lead to the recruitment of a histone deacetylase. To investigate this possible mechanism of transcriptional repression, we have focused on ZBTB33 (also known as Kaiso), a member of the zinc finger and BTB (ZBTB) family of site-specific transcription factors, which has been shown in vitro to preferentially bind a DNA motif containing methylated CpG dinucleotides (Figure 1). Among the ZBTB family are three proteins, ZBTB33, ZBTB4, and ZBTB38; all of which have been shown in vitro to use three tandem C2H2 zinc finger domains to preferentially bind methylated DNA [7]. Specifically, Kaiso has been shown in vitro to bind a motif containing CGCG (Figure 1A), but only when both cytosine residues are methylated [8]. Studies show that all three of Kaiso’s C2H2 zinc finger domains are required for its binding to this methylated motif [8]. Other in vitro results also suggest Kaiso is capable of binding to an unrelated unmethylated motif, TCCTGCNA [9]. However, the relationship between Kaiso binding motifs and DNA methylation has not yet been investigated on a genome-wide scale in human cells.

A model has been proposed in which Kaiso binds to methylated DNA in a promoter region, recruits components of the NCoR histone deacetylase complex, and represses transcription (Figure 1B). This model is based in part on analysis of the MTA2 promoter. Yoon et al. [10] showed that Kaiso can bind in vitro to a methylated version of the sequence GGCGCGCGAGTCTTTGGGGCGCG, which is found within the CpG island promoter of the MTA2 gene. Using primers specific for methylated DNA, they also showed that this sequence is methylated in HeLa cells and they performed ChIP experiments to show that Kaiso binds this site before, but not after, cells are treated with 5aza-dC, a drug which reduces DNA methylation levels. They show that Kaiso interacts with the NCoR complex and demonstrate that Kaiso fused to the GAL4 DNA binding domain can repress transcription of a luciferase reporter containing a GAL4 binding site. Finally, they show that treating cells with siRNAs to Kaiso, 5aza-dC or the HDAC inhibitor TSA can increase MTA2 RNA. Taken together, they proposed a model in which Kaiso binds to the methylated MTA2 promoter and represses transcription by reducing levels of acetylated histones near the start site. We wished to test this model on a genome-wide scale and therefore have analyzed Kaiso binding in normal and cancer human cell lines, performed motif analyses of the in vivo binding sites, and investigated the relationship between Kaiso binding, DNA methylation, modified histones, and gene expression levels. Our results suggest that, in contrast to in vitro studies, in cells Kaiso binds to highly active, unmethylated promoters.

## Results

### Identification and characterization of Kaiso-binding sites

We began by downloading the sequenced tags for all Kaiso ChIP-seq datasets from the UCSC browser (from the ENCODE consortium); two replicate Bowtie-mapped ChIP-seq datasets were downloaded for GM12878, K562, A549, HepG2, HCT116, and SK-N-SH cells. Peaks were called for each replicate in each cell type using Sole-Search [11,12]; see Table 1. To determine the reproducibility of the data, an overlap analysis was performed by truncating both replicates to the same peak number and comparing the top 40% of replicate 1 to the entire set of peaks of replicate 2 (and vice versa, which is indicated as the reciprocal overlap in Table 1). Of the six cell lines for which Kaiso ChIP-seq data is available, we found that the data from GM12878, K562, and A549 cells passed ENCODE standards for biological replicates (number of peaks in each set must be within a factor of two and after truncation of peak sets to the same number, 80% of the top 40% of the peaks from one replicate must overlap with the entire set of peaks of the other replicate); see Landt et al. [13]. Although, the HepG2 datasets are close to ENCODE standards (numbers of peaks are within a factor of two and there is a 71% overlap using the 40% rule), the Kaiso ChIP-seq datasets from HCT116 and SK-N-SH cells clearly do not pass the quality standards [13], due in large part to the great difference in the number of peaks identified in the two replicates of each dataset. In our studies we have focused on the Kaiso datasets from GM12878 and K562 cells.

**Table 1:** **Sequencing and peak metrics for ENCODE Kaiso ChIP**-**seq datasets**

	**GM12878**	**K562**^**1**^	**A549**	**HepG2**	**HCT116**	**SK**-**N**-**SH**
Unique reads, Rep1	16,619,899	26,444,144	34,298,781	12,992,054	20,455,526	20,984,176
Sole-Search peaks, Rep1	2,396	11,257	14,414	1,560	8,813	12,290
Median peak tag height Rep1	24	25	25	26	21	23
Unique reads, Rep2	14,307,805	19,111,076	34,105,543	18,274,016	4,305,814	10,093,008
Sole-Search peaks, Rep2	2,784	15,395	11,559	2,342	936	2,789
Median peak tag height, Rep2	21	21	23	25	19	21
40% overlap (reciprocal overlap)	96% (97%)	83% (92%)	85% (81%)	66% (71%)	98% (94%)	65% (81%)
Unique reads, merged Reps	30,621,961	44,860,842	67,432,203	31,180,904	24,667,177	30,833,060
Sole-Search peaks, merged Reps	12,543	18,651	42,862	2,529	12,325	22,172
Median peak tag height, merged	20	34	27	30	23	25
High-confidence peaks	1,648	3,082	7,658	757	902	2,675
Median peak tag height	34	28	30	44	78	37
IDR peaks	2,144	3,285	7,152	2,879	4,325	N/A^2^

For each dataset, two biological replicates of Bowtie-aligned .bam files were downloaded from the UCSC genome browser, and peaks were called using Sole-Search. The replicate with the most Sole-Search-called peaks was truncated so that both replicates contained the same number of peaks. Replicates were then compared by ENCODE standards using the 40% overlap rule (overlap percentage shown). Also shown are peak metrics for high-confidence Kaiso peaks in each cell line.

^1^ In the K562 dataset, amplified genomic regions were discovered to contain numerous false peaks that were not all removed by the peak-calling program; these peaks were removed from the list of high-confidence K562 peaks (see Additional file 2: Figure S2). The removed peaks had very high peak tag numbers because the amplified regions were over-sequenced. As a result of removing these false positive peaks, the median peak tag height of the high-confidence peaks is lower than the median peak tag height of the merged peak set. ^2^ At the time of paper submission, IDR peaks had not been called for Kaiso in SK-N-SH.

**Figure 2:**
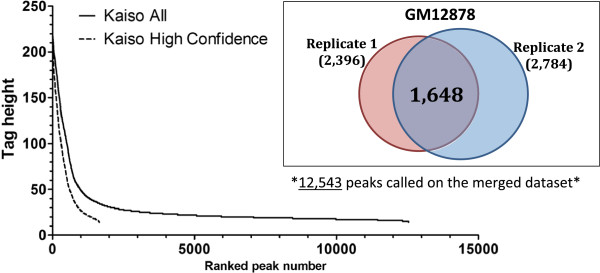
**Identification of high**-**confidence Kaiso peaks in GM12878 cells.** The set of 12,543 Kaiso peaks identified in GM12878 cells using the merged replicate datasets (solid line) and the set of 1,648 high-confidence Kaiso peaks (dashed line) is plotted as tag height versus ranked peak number. The inset shows a Venn diagram of the overlap between the two Kaiso ChIP-seq replicates in GM12878 cells. The total number of peaks in each replicate is represented in parentheses; 1,648 high-confidence peaks were found to be present in both replicates.

After determining the quality of the Kaiso ChIP-seq datasets, the next step was to select a defined peak set for further analysis. Because of the nature of the ChIP-seq assay, there is not a discrete number of peaks for any transcription factor. Rather, as more reads are acquired, more and more peaks are identified. The peaks that are only identified when large numbers of reads are used tend to be much smaller than the median height of the peaks identified when less than 10 million reads are analyzed. The issue of small peaks is especially problematic when two ChIP-seq replicate datasets (each of which having more than 10 million reads) are merged. As shown in Figure 2 for Kaiso ChIP-seq data from GM12878 cells, there is a clear inflection point (separating a minority of large peaks having a peak height about 40 from 90% of the peaks having a tag height below 40) when peak height is plotted versus ranked peak number. Although some of the small peaks may be of biological importance, we have found that in many ChIP-seq analyses most of the small peaks are not reproducible. Inclusion of false-positive (non-reproducible) small peaks can greatly skew motif analyses and muddy insights into the relationship of a particular DNA-binding protein and transcriptional regulation. Therefore, we have chosen to use only high-confidence Kaiso peaks in our analyses. We define high-confidence peaks as peaks present in both replicates, regardless of peak height. As shown in Figure 2 (inset), although 12,543 peaks were identified in GM12878 cells using the merged replicates, only 1,648 peaks were identified as common to both replicates (each of which had approximately 2,400 to 2,800 peaks). The peak height versus peak rank of the 1,648 high-confidence Kaiso peaks is plotted in Figure 2A (dashed line), for comparison with all peaks identified using the merged datasets (solid line). Although some small peaks with a tag height below 40 were retained because they were reproducibly identified in both replicates, most of the small peaks were removed using this analysis. We next identified a set of ‘high-confidence’ Kaiso-binding sites for each cell type using this method (Table 1); these peaks sets were used for further analyses in our studies. We also note that these Kaiso datasets have also been analyzed by the ENCODE consortium using the IDR program, which provides an estimate of the number of reproducible peaks between two replicates [14]. Although for the three high quality datasets (GM12878, K562, and A549), the IDR-called peaks are similar in number to the high-confidence peaks we have identified, we note that there will be some difference in the exact peaks which are retained using the different methods. Because IDR uses the merged dataset to call a final peak set, large peaks that are present in only one dataset may be included whereas certain small, but reproducible, peaks may be excluded. For the three datasets that do not pass quality standards (HepG2, HCT116, and SK-N-SH), the IDR-called peaks may contain many false positives; if these datasets are used by the community, we recommend that only the smaller set of high- confidence Sole-Search-called peaks be used. Genomic coordinates of all high-confidence peak sets for all Kaiso datasets can be found in Additional file 1.

### Testing a model of Kaiso-mediated transcriptional repression

As described above, Kaiso has been shown to bind to methylated DNA and has been suggested to function as a transcriptional repressor via recruitment of HDACs via interaction with the NCoR and SMRT complexes [8,10,15-18]. If true, this could provide a mechanistic explanation for a link between promoter methylation and transcriptional repression. However, these studies were performed in vitro and/or focused on small sets of genes. To test this model on a genome-wide scale, we have examined the location of Kaiso binding sites asking the following questions: 1) Are Kaiso binding sites located in promoter regions? 2) What is the epigenetic status of the regulatory regions bound by Kaiso? and 3) what is the DNA methylation status of Kaiso binding sites?

**Figure 3:**
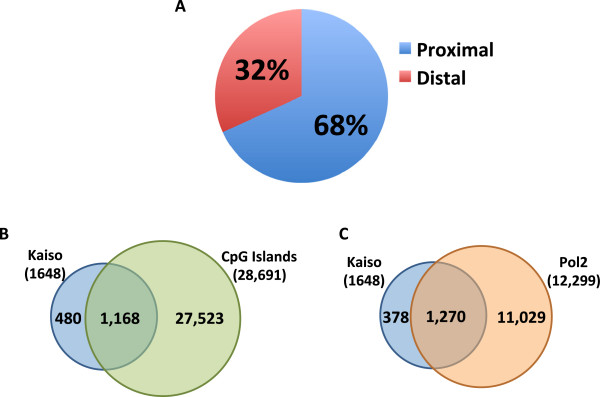
**Kaiso binds to GC**-**rich promoters that are also bound by Pol2.** (**A**) Shown is the percentage of Kaiso peaks in GM12878 cells located within 1 kilobase of a Refseq transcription start site (proximal, blue), and those located further than 1 kilobase away from a transcription start site (distal, red). (**B**) Shown is the percentage of Kaiso peaks in GM12878 cells that are found within CpG islands. (**C**) Shown is the percentage of Kaiso peaks in GM12878 cells that overlap the top-ranked set of Pol2 peaks (see Additional file 2).

To investigate the mechanism(s) by which Kaiso functions on a genome-wide scale, we first determined the location of the high-confidence Kaiso ChIP-seq peaks in GM12878 cells relative to transcription start sites using the Sole-Search location analysis tool. We found that approximately 70% of high-confidence Kaiso peaks bind within 1 kb of a transcription start site of a Refseq gene (Figure 3A). A similar percentage of Kaiso peaks was also found to bind within CpG islands (Figure 3B), indicating that Kaiso prefers to bind at GC-rich promoters. To determine if Kaiso bound to promoters that were also bound by RNA polymerase II (Pol2), we first identified a set of top-ranked Pol2 peaks (Additional file 2A). To do so, we downloaded the Pol2 ChIP-seq tags from GM12878 from the ENCODE data on the UCSC browser and called peaks using Sole-Search, identifying approximately 50,000 peaks. Pol2 is known to be involved in looping [19,20] and it is likely that many of the very small peaks are due to protein-protein interactions. To enrich for those peaks that represent direct binding of Pol2 to transcription start sites, we chose a set of top-ranked peaks for downstream analysis. The peaks chosen represent the top 20% of the Pol2 peaks and the number of tags of the lowest ranked peak chosen is 2.6-fold over the median value of the peaks not chosen. Pol2 peaks chosen for analysis were compared to those discarded for their proximity to known transcription start sites. The top Pol2 peaks are highly enriched at transcription start sites whereas the lower background peaks are primarily promoter distal (Additional file 2B). We then overlapped Kaiso peaks with the selected top-ranked Pol2 peaks; the majority of Kaiso binding sites overlap the top 20% of Pol2 peaks (Figure 3C), indicating that the promoters bound by Kaiso are either active or poised for transcription because they have robust Pol2 signals.

**Figure 4:**
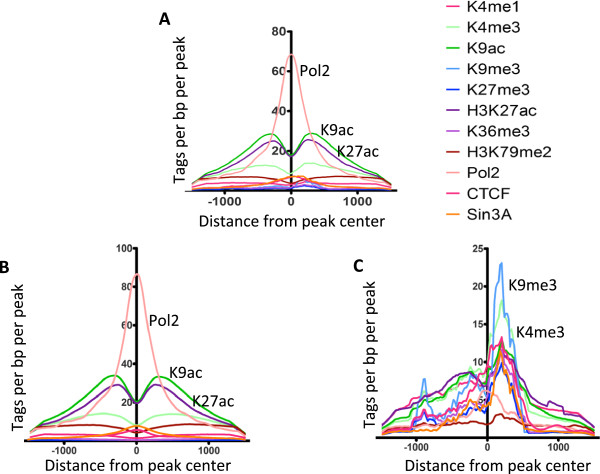
**Epigenomic profiles of Kaiso binding sites in GM12878 cells.** The density of ChIP-seq tags for several histone modifications and transcription factors is plotted relative to subsets of Kaiso binding sites in GM12878; (**A**) all high-confidence Kaiso peaks, (**B**) high-confidence Kaiso peaks that overlap with top-ranked Pol2 peaks, (**C**) high-confidence Kaiso peaks that do not overlap with top-ranked Pol2 peaks.

To characterize the epigenetic profiles of the Kaiso binding sites, tag density plots were created to identify enrichments of specific histone modifications proximal to Kaiso binding sites. Tag density plots provide a more accurate view of the relationship of different ChIP-seq datasets than do simple peak overlap analyses. This is because all tags are used in these analyses, eliminating any user-selected cut-offs. ChIP-seq datasets for the various histone modifications, Pol2, CTCF, and Sin3A in GM12878 cells were downloaded from the UCSC browser and compared to the Kaiso peak genomic coordinates. Consistent with the overlap analysis of Kaiso peaks and Pol2 peaks shown above, Pol2 ChIP-seq tags were highly enriched at Kaiso peaks (Figure 4A). Analysis of the epigenetic modifications of the nucleosomes surrounding the Kaiso peaks revealed an enrichment of active histone modifications at Kaiso binding sites. Specifically, there is a large enrichment of acetylated histone H3, lysine 9: the mark most predictive of actively-transcribed genes [6]. Another histone mark associated with open chromatin, acetylated histone H3 lysine 27, was also highly enriched at Kaiso peaks. These results suggesting that most Kaiso binding sites are at active promoters were performed using the entire set of Kaiso binding sites. However, as shown above, 20% of the Kaiso binding sites did not overlap with robust Pol2 peaks. It is possible that the Kaiso bound to this subset of sites may be involved in a different mode of transcriptional regulation. Therefore, we separated the Kaiso peaks into those that overlapped with Pol2 versus those that did not overlap Pol2. The Kaiso peaks that overlapped with Pol2 had essentially the same profile as the entire set of Kaiso peaks (Figure 4B). However, the 378 Kaiso peaks that did not overlap with the top-ranked Pol2 peaks had a very different epigenetic profile; these peaks had very low enrichments for the active histone modifications (Figure 4C). It should be noted that the non-Pol2-bound Kaiso sites did not have a robust enrichment for Sin3A, a component of the NCoR HDAC complex. The highest enriched modification for this set of peaks is H3K9me3, perhaps suggesting a link between Kaiso binding to methylated DNA and transcriptional repression via H3K9me3 at these sites; this subset of Kaiso-binding is further analyzed as a separate subclass below in the DNA methylation studies.

**Figure 5:**
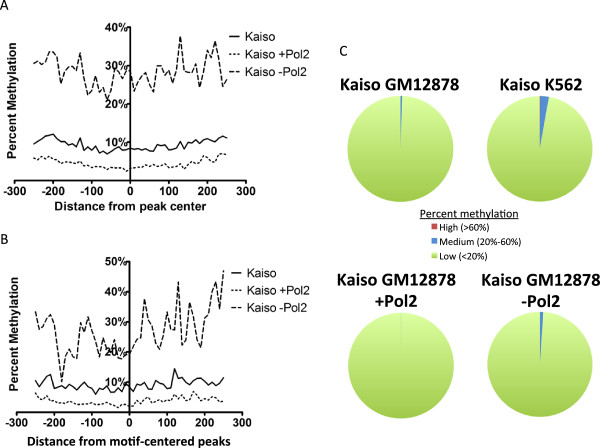
**DNA methylation analysis of Kaiso peaks.** (**A**) The average percent DNA methylation in the sequences surrounding the Kaiso binding sites (centered on the middle of the peak) was determined and plotted across all high-confidence Kaiso peaks (solid line), high-confidence Kaiso peaks overlapping Pol2 (short dashed line), and high-confidence Kaiso peaks not overlapping Pol2 (long dashed line). (**B**) A similar analysis was performed as in panel (**A**) except that only the subsets of high-confidence Kaiso peaks containing the 10 bp Kaiso motif were used and the regions were centered on the Kaiso motif. (**C**) Pie charts depicting the methylation percentage of all CGCG motifs in various sets of Kaiso peaks.

As a third method of characterization of the Kaiso binding sites, we determined if Kaiso binds to promoters having high levels of DNA methylation. Using whole genome bisulfite sequencing (WGBS) and reduced representation bisulfite sequencing (RRBS) data from GM12878 cells, we determined the DNA methylation profile of the sequences surrounding the Kaiso binding sites. As shown in Figure 5A, the set of all Kaiso peaks and the set of Kaiso peaks that overlap Pol2 peaks both have very low DNA methylation profiles. Thus, the majority of the genomic regions bound by Kaiso in GM12878 cells are GC-rich promoters that have low levels of DNA methylation and high levels of acetylated histones. Although Kaiso peaks not overlapping Pol2 were found to have slightly higher methylation, this level of methylation (approximately 30%) is similar to that found at the center of CTCF peaks, a factor shown to bind to unmethylated DNA [21].

If Kaiso binds to a short region of highly methylated DNA that is not exactly in the middle of a peak having an overall low level of DNA methylation, then it is possible that a relationship between Kaiso binding and DNA methylation is obscured when the analyses are centered on the middle of the genomic regions identified by the peak-calling program. To alleviate this concern, the DNA methylation analyses can be centered on the Kaiso binding motif. Kaiso has been shown to bind to a methylated motif TCTCGCGAGA in vitro and in vivo [18], and an unmethylated motif TCCTGCNA in vitro. To identify an enriched motif within Kaiso binding sites, a motif analysis was performed using Hypergeometric Optimization of Motif EnRichment (HOMER) software [22] to identify strings of nucleotide sequences that are enriched within Kaiso peaks. HOMER produces separate output files for de novo identified motifs and known motifs identified in the peak set. HOMER’s de novo motif algorithm identified the previously published ‘methylated’ Kaiso motif TCTCGCGAGA as the most highly represented motif in the 1,648 high-confidence Kaiso peaks, in the 1,270 Pol2-overlapping peaks, and in the 378 non-Pol2-overlapping peaks. 36 to 43% of the Kaiso binding sites contained a match to this motif. However, the previously identified ‘unmethylated’ in vitro motif TCCTGCNA was not enriched in the Kaiso peak sets (Table 2). Absence of Kaiso binding to the TCCTGCNA motif has also been reported by Ruzov et al. [23], who performed ChIP-seq of a tagged Kaiso protein; however, the significance of these results was not clear because mouse and Xenopus Kaiso was studied in human HEK293 cells. If Kaiso is in fact binding methylated DNA, it is likely doing so via the Kaiso methylated motif (because the unmethylated motif does not contain a CpG). Therefore, we selected the subset of Kaiso peaks containing the TCTCGCGAGA motif for further analysis (Additional file 3). Because there is a wide distribution of the location of this motif relative to the center of Kaiso peaks, this could perhaps mute the observation of a peak of DNA methylation within the actual motif. To correct for this possibility, we centered Kaiso peaks on the TCTCGCGAGA motif and repeated the methylation analysis (Figure 5B). This analysis again did not show a high enrichment of DNA methylation even when focusing only on the peaks that contain the TCTCGCGAGA motif. However, these analyses use a 10 bp sliding window and therefore it is possible that the majority of the CpG dinucleotides in the Kaiso peaks are unmethylated (lowering the overall average DNA methylation value of any 10 bp window) but that the central CGCG of the recognition motif is methylated. To determine if this is true, we identified all Kaiso peaks having a central CGCG (Additional file 3); see Table 2 for the percentage of peaks containing a core CGCG. We then determined the methylation status of each occurrence of a CpG dinucleotide in all Kaiso peaks containing a central CGCG (Additional file 4). Figure 5C shows the fraction of CGCG motifs in the Kaiso peak sets that contain high (> 60%), medium (20% to 60%), or low (< 20%) levels of methylation. Of the 4,781 CGCG motifs containing greater than 3× sequencing coverage identified within high-confidence Kaiso peaks, 26 motifs contained medium levels of DNA methylation with the highest level being 43%. Most of the motifs containing medium levels of methylation are found in the subset of Kaiso peaks not overlapping Pol2.

**Table 2:** Motif analysis of Kaiso binding sites

	**KaisoHC GM12878** (**1**,**648 sites**)	**KaisoHC**+**Pol2HC GM12878** (**1**,**270 sites**)	**KaisoHC**-**No Pol2HC GM12878** (**378 sites**)	**KaisoHC K562** (**3**,**082 sites**)
TCTCGCGAGA	43%	42%	36%	29%
CGCG	76%	85%	35%	47%
TCCTGCNA	5%	6%	4%	5%

The percentage of peaks containing each motif displayed on the left side of the chart was calculated for several subsets of Kaiso peaks in GM12878 and K562 cells. The total number of peaks in each subset is shown in parentheses.

### Characterization of Kaiso binding sites in cancer cells

The analyses of Kaiso binding sites described above were performed using GM12878 cells, which is an EBV immortalized normal lymphoblastoid cell line. Because Kaiso binds to promoter regions, it is possible that the lack of correlation between Kaiso binding and DNA methylation is simply due to the fact that most CpG island promoters are hypomethylated in normal cells. In contrast, in cancer cells although the majority of the genome is hypomethylated, many promoters become hypermethylated. Thus, it is possible that there are more methylated TCTCGCGAGA motifs in cancer cells, providing a platform for Kaiso to bind to methylated DNA. To determine if Kaiso binds to methylated DNA in a cancer cell, we analyzed Kaiso binding sites in the myeloid leukemia cell line, K562. Kaiso was determined to be similarly expressed in GM12878 and K562 by Western blot and by RNA-seq data (see Methods). We first identified a set of Kaiso binding sites that were present in both replicates of the K562 Kaiso ChIP-seq datasets. The number of these sites was much higher than the number of GM12878 Kaiso binding sites and therefore we performed further inspection of the K562 peaks. We noted that a much lower percentage of the K562 Kaiso peaks contained the Kaiso motif than did the GM12878 peaks (12% versus 43%), suggesting that the K562 peak set may contain a large number of false positive peaks. K562 cells are cancer cells and have a number of highly-amplified genomic regions. Although our peak-calling program has features that remove many of the false positive peaks due to genomic amplifications [11,12], we observed that a large number of peaks from K562 cells were in the amplified regions. We removed these amplified regions from our analyses and the peak set was reduced to approximately 3,082 peaks, approximately 30% of which contained a TCTCGCGAGA motif (Additional file 5). This adjusted peak set was used for all downstream analyses as the high-confidence Kaiso peaks in K562. Additional file 6 contains the genomic coordinates of the amplified regions in K562 and the Kaiso peaks before and after removal of these amplified regions.

To determine if Kaiso binds to methylated promoters in K562 cells, we selected the set of Kaiso peaks that contained a CGCG motif and determined the methylation percentage of each of the CpG dinucleotides. In this case, we used RRBS data for DNA methylation analysis because WGBS data is not available for K562 cells. The genomic coverage is lower in the K562 data than in the GM12878 data; the number of CpGs with 3× coverage or greater in the K562 RRBS dataset is 1 million, compared to 45.6 million in the GM12878 methylation data for which we could use a combination of WGBS and RRBS. Therefore, with a 3× coverage cut-off, we could only analyze 1,069 CGCG motifs in the K562 Kaiso peaks. Of these, 28 peaks contained medium levels of DNA methylation and only two peaks contained high levels of methylation (Figure 5C); a snapshot of the chromosomal region containing the highest methylated CGCG motif (63% methylated) is shown in Additional file 7.

### Cell type-specific binding of Kaiso

**Figure 6:**
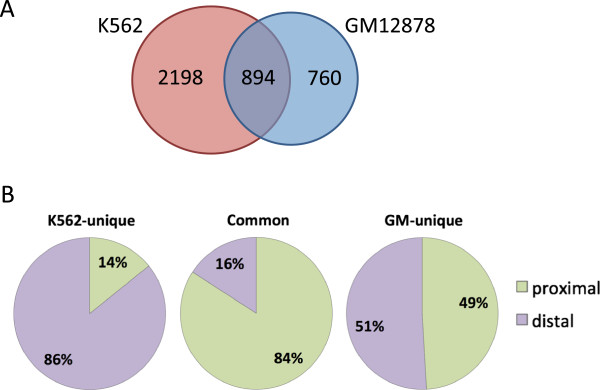
**Characterization of cell type**-**specific Kaiso binding sites.** (**A**) High-confidence Kaiso peaks in GM12878 and K562 were overlapped to identify common peaks and cell type-specific peaks between the two datasets. (**B**) The common and cell type-specific Kaiso peaks were analyzed for their position relative to the start site of transcription of the set of Refseq genes; sites within +/- 1 kb of a start site were classified as promoter proximal whereas all other sites were classified as promoter distal.

**Figure 7:**
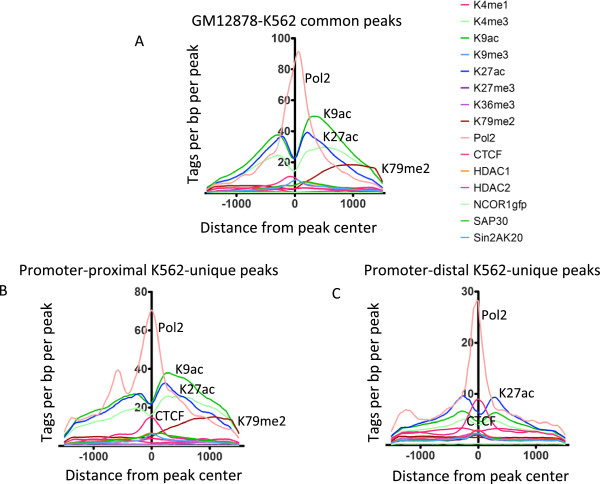
**Epigenomic profiles of Kaiso binding sites in K562 cells.** The density of ChIP-seq tags for several histone modifications and transcription factors is plotted relative to subsets of Kaiso binding sites in K562 cells; (**A**) peaks common between K562 and GM12878 cells, (**B**) promoter proximal peaks unique to K562, (**C**) promoter distal peaks unique to K562.

During our analysis of the methylation status of Kaiso binding sites, we noted that there are many more Kaiso binding sites in K562 cells than in GM12878 cells. This suggested that there might be cell type-specific binding of Kaiso. To test this hypothesis, we performed an overlap analysis of the K562 and GM12878 Kaiso high-confidence peak sets and found that 894 peaks were shared in the two cell types, but that 760 and 2,198 sites were uniquely bound by Kaiso in GM12878 and K562 cells, respectively (Figure 6A). A location analysis revealed that the common sites were highly specific for promoter regions (Figure 6B). In contrast, only half of the sites specific to GM12878 were at promoter regions whereas the majority of the sites specific to K562 were located outside of promoter regions. It is possible that Kaiso may have a different role in transcriptional regulation when bound near versus far from transcription start sites. Therefore, we determined the epigenetic profiles of the promoters bound by Kaiso in both GM12878 and K562 (mostly promoter proximal) and of the sets of Kaiso binding sites unique to K562 cells that were promoter proximal versus promoter distal. The promoter proximal peaks common between K562 and GM12878 are enriched for Pol2, the active marks H3K9Ac and H3K27ac, and the transcription elongation mark H3K79me2 (Figure 7A); this evidence suggests that these regions are actively transcribed promoters. Very similar epigenetic profiles are seen for the K562-specific Kaiso-bound promoters (Figure 7B), suggesting that all promoters bound by Kaiso in K562 cells (common or cell-type specific) are actively transcribing. Interestingly, the 1,890 promoter distal peaks unique to K562 are also enriched for Pol2 and acetylated histones (Figure 7C). The overall level of enrichment for these active marks is lower at the distal sites than at known promoters. This could be due either to the presence of a small number of bona fide promoters of genes or non-coding RNAs that are not in the Refseq dataset or perhaps an indication of chromosomal looping between active promoters and a distal site bound by Kaiso. To address this issue, we overlapped the set of K562-unique promoter distal Kaiso peaks with the top 20% of the Pol2 peaks from K562 cells. We found that 24% of these Kaiso peaks overlap with the top-ranked Pol2 peaks, suggesting that perhaps a quarter of the 1,890 Kaiso K562 binding sites classified as distal are at promoters that are not annotated in Refseq dataset. Most importantly, no subset of Kaiso peaks in K562 was determined to have an enrichment for components of the NCoR or SMRT histone deacetylation complexes, contrary to what would be expected based on previously published findings [10,18].

**Figure 8:**
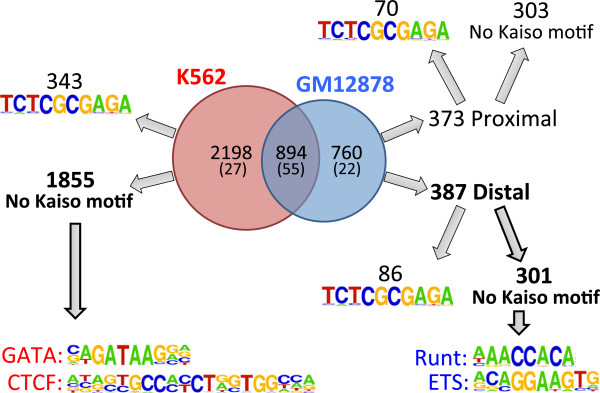
**Motif analysis of cell type**-**specific Kaiso binding sites.** Kaiso peaks from GM12878 and K562 were compared to identify common and cell type-specific binding sites (the median tag height is shown in parentheses). GM12878- and K562-unique peaks were divided into two sets of peaks, those that contain and those that lack a Kaiso motif; GM12878-unique peaks were further separated into promoter proximal and promoter distal sets. A motif analysis was then performed for each set of peaks, identifying motifs for the GATA and CTCF families of transcription factors in the K562-unique peaks and motifs for the ETS and Runt families of transcription factors in distal GM12878 peaks (see also Additional file 8).

**Figure 9:**
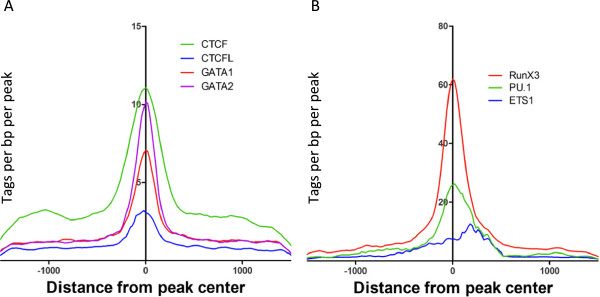
**Testing bioinformatic predictions of transcription factor co**-**localization.** The density of ChIP-seq tags for the factors identified by motif analysis in Figure 8 were plotted relative to (**A**) non-motif containing K562 Kaiso peaks, and (**B**) promoter distal, non-motif containing GM12878 Kaiso peaks.

As shown above, 46% of the Kaiso peaks in GM12878 cells and 71% of the Kaiso peaks in K562 cells are cell type-specific. Although these sites are reproducible (having been identified in both replicates for each cell type) the median tag height for the cell type-specific binding sites is lower than for the sites occupied by Kaiso in both GM12878 and K562 cells (tag height is shown in parentheses in Figure 8). This suggests that Kaiso binding at the cell type-specific sites may occur by a different mechanism than at sites common to both cell types. There are several mechanisms that can mediate cell type-specific binding. One mechanism is that Kaiso could be recruited to the genome by an interaction with cell type-specific DNA-binding factors at sites that contain no or weak matches to the Kaiso motif. To examine the possibility that Kaiso is being recruited to sites in K562 or GM12878 cells via a cell type-specific binding partner, we first determined if the cell type-specific peaks contained the Kaiso motif. For both GM12878 and K562 cells, we found that approximately 80 to 85% of the cell type-specific Kaiso peaks lacked a TCTCGCGAGA motif. To test the possibility that another site-specific factor aided in the recruitment of Kaiso to the non-motif containing cell type-specific sites, we performed a motif analysis using HOMER. Strikingly, we found that a GATA motif corresponded to five of the top eight most enriched motifs in the set of K562-unique peaks that did not have a Kaiso motif; the other 3 motifs corresponded to reported motifs for CTCF or CTCFL (Figure 8; Additional file 8A). We performed a similar analysis for the GM12878-specific Kaiso peaks except that we separately analyzed promoter proximal and distal sites not containing a Kaiso motif (this separation was not necessary for K562 because the majority of the sites were distal). The proximal regions did not return significant results, likely due to the large number of conserved sequences found at all promoter regions. Separate analysis of the distal subset, which is more similar to the K562 unique peaks, eliminated problems that occur with motif analyses of promoter regions. We found that motifs for ETS and Runt family members are enriched in the GM12878-specific, distal, non-Kaiso motif set (Figure 8; Additional file 8B). To determine if Kaiso co-localizes with GATA or CTCF family members in K562 cells or with ETS or Runt family members in GM12878 cells, we downloaded the GATA1, GATA2, CTCF, and CTCFL ChIP-seq data from K562 cells and the ETS1, PU.1, and RUNX3 ChIP-seq data from GM12878 cells from the UCSC Genome Browser and created tag density plots relative to Kaiso peaks (Figure 9). In K562, we found that GATA1, GATA2, CTCF, and CTCFL were all enriched at the center of Kaiso peaks with CTCF and GATA2 being the most enriched (Figure 9A). Interestingly, a previous study has shown that Kaiso can interact with CTCF [24]. When comparing binding of ETS and Runt family members to Kaiso sites in GM12878, we again found that members of both families co-localized with Kaiso, with RUNX3 and PU.1 having the highest enrichment (Figure 9B).

**Figure 10:**
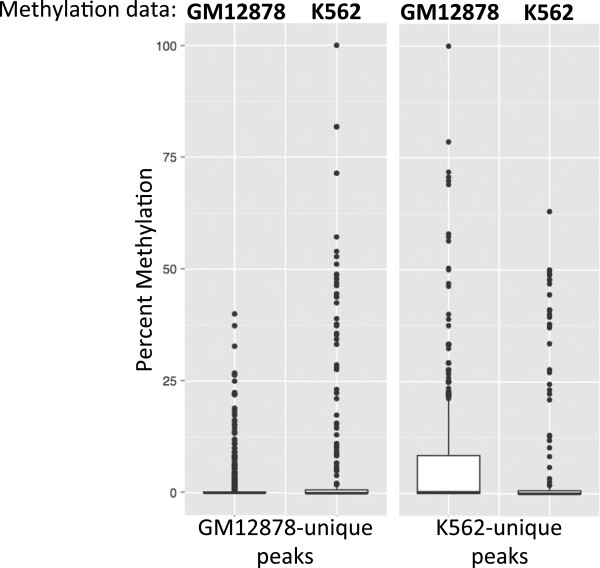
**Methylation of cell type**-**specific CGCG motifs in different cell lines.** The percent methylation of individual CGCG motifs within GM12878-unique and K562-unique peaks was calculated using GM12878 WGBS and RRBS data and K562 RRBS data, and graphed as box plots showing differences in methylation between cell types.

**Figure 11:**
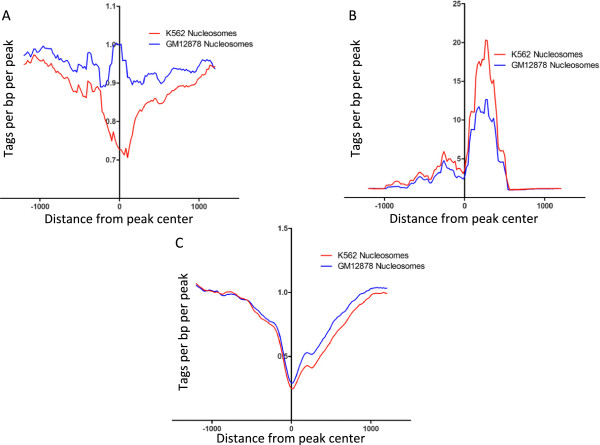
**Nucleosome density of cell type**-**specific Kaiso peaks in GM12878 and K562.** Nucleosome positioning data from MNase-seq was used and plotted as the average density of nucleosomes relative to Kaiso peaks in **A**) GM12878, **B**) K562, and **C**) common sites.

A second mechanism that can contribute to cell type-specific binding of a ubiquitously expressed transcription factor is site availability. For example, high levels of DNA methylation at a promoter results in a nucleosome-dense, silenced region that is not accessible to transcription factors. Thus, a promoter that has the potential to be a Kaiso target (for example, because it contains a Kaiso motif and is bound by Kaiso in GM12878 cells) may not be bound by Kaiso in K562 cells if the promoter is highly methylated and densely packed by nucleosomes. To address whether differences in DNA methylation are responsible for cell type-specific binding to promoters in GM12878 versus K562 cells, we determined the methylation status in GM12878 cells of the K562-specific Kaiso peaks and the methylation status in K562 cells of the GM12878-specific peaks (Figure 10). We found that GM12878-unique sites had higher overall levels of methylation in K562 cells and K562-unique sites had higher overall levels of methylation in GM12878, suggesting that at least a portion of the cell type-specificity of Kaiso binding is due to exclusion from promoters due to DNA methylation. Next, we looked at the density of nucleosomes relative to GM12878- and K562-unique and common binding sites. MNase-seq data produced by ENCODE was downloaded from the UCSC genome browser, and was used to determine the nucleosome density of Kaiso binding sites. Tag density plots show that sites common between GM12878 and K562 contain low levels of nucleosome density, particularly at the center of the peak (Figure 11B). Conversely, K562-unique peaks are nucleosome-free in K562 cells, but are occupied in GM12878 (Figure 11A). This suggests that the occupancy of a nucleosome at a potential binding site in GM12878 restricts the binding of Kaiso to that region, contributing to the cell-type-specificity of Kaiso binding. GM12878-unique sites show a slight nucleosome occupancy increase in K562 cells, but overall, very similar nucleosome density is observed in both cell lines at these sites. This suggests that nucleosome occupancy plays a lesser role in the cell type-specific Kaiso binding in GM12878 cells (Figure 11C), perhaps due to less overall DNA methylation at promoters in GM12878 cells. Together, the DNA methylation and nucleosome occupancy results suggest that chromatin structure plays a partial role in the cell type-specificity binding of Kaiso between GM12878 and K562.

**Figure 12:**
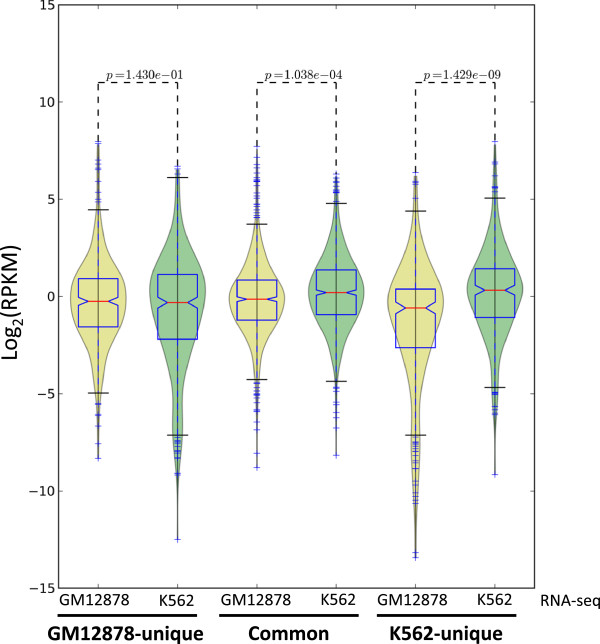
**Expression of cell type**-**specific Kaiso**-**bound promoters in GM12878 and K562.** RPKM values from RNA-seq experiments in GM12878 and K562 cells were used to create box plots showing expression of cell type-specific and common Kaiso-bound genes.

Lastly, we have used RNA-seq data from GM12878 and K562 to examine the relationship between Kaiso binding and gene expression. We identified the genes nearest to the Kaiso binding sites that were in common between the GM12878 and K562 Kaiso ChIP-seq datasets and the genes that had Kaiso bound to their promoter regions only in GM12878 or only in K562 cells. We then determined the expression levels of these three sets of genes in both GM12878 and K562 cells (Figure 12). We found that of these three gene sets, the most highly expressed was the set of genes bound by Kaiso in both GM12878 and K562 cells. Interestingly, the GM12878-specific Kaiso targets are more highly expressed in GM12878 cells than in K562 cells and the K562-specific Kaiso targets are more highly expressed in K562 cells than in GM12878 cells. Thus, we see a positive correlation between gene expression and Kaiso binding.

## Discussion

The purpose of this study was to test the previously proposed model that Kaiso functions as a DNA methylation-dependent transcriptional repressor that causes local depletion of acetylated histones near its binding sites. In our studies, we bioinformatically analyzed genome-wide binding of Kaiso in normal and cancer cells, examined the epigenetic profiles of Kaiso-bound promoters, and determined the percentage of DNA methylation of CpGs bound by Kaiso. Although the analyses presented in this study focused on only two cell lines, we have also examined the relationship between Kaiso binding sites, Pol2, and histone modifications in HCT116, A549, and SK-N-SH cells (see Table 1 for the number of high- confidence Kaiso peaks analyzed in each dataset). In each cell type, Kaiso is associated with active promoters (unpublished data). In addition, we performed whole genome bisulfite sequencing of HCT116 and found that Kaiso binding sites are not methylated; specifically, out of 3,051 CGCG motifs within the high-confidence HCT116 Kaiso-binding sites, 3,036 (99.5%) had methylation levels less than 20% (unpublished data). Thus, results from all tested cell lines are similar but do not support the previously proposed model. Specifically, we find that Kaiso does not bind to methylated DNA and that it is associated with highly acetylated, actively transcribed promoters. Our results are consistent with those presented in Factorbook [25], an online repository that hosts analyses of ENCODE transcription factor ChIP-seq data. However, due to the large scale of that project, individualized analyses were not performed for each factor. For example, the Factorbook analysis of motifs found in Kaiso binding sites included only the top 500 peaks and did not specifically search for the non-methylated motif to which Kaiso has been shown to bind in vitro. Another difference is that Factorbook uses the ENCODE IDR peak sets which, as we describe above, do not represent the same peaks as our high confidence peaks that are found in both ChIP-seq replicates for a given cell type. In addition, our studies included a thorough analysis of cell type-specific binding patterns, motifs, and nucleosome occupancy of Kaiso sites. Finally, Factorbook did not include an analysis of DNA methylation, which is the central theme of our study.

Several different groups have used in vitro gel shift assays to demonstrate that Kaiso binds to the motif TCTCGCGAGA when both CpGs in the motif are methylated [7-10,26]. In addition, Bartels et al. performed an unbiased in vitro screen for methyl DNA-binding proteins and identified Kaiso using mass spectrometry analysis of the proteins captured in the screen [27]. Also, a recent study has solved the structure of Kaiso bound to methylated DNA [28]. Clearly, Kaiso does bind to methylated DNA in vitro. However, the vast majority of in vivo Kaiso binding sites have very low levels of DNA methylation. Visual inspection of the rare sites that have high DNA methylation revealed that Kaiso has the ability to bind methylated DNA in vivo, but that the Kaiso peaks at those sites are among the lowest enriched binding sites. In fact, a plot of peak score versus percent DNA methylation clearly shows that the strongest Kaiso sites have very low levels of DNA methylation (Additional file 9). These data do not support the in vitro studies that indicate that Kaiso prefers to bind to the TCTCGCGAGA motif when both CpG dinucleotides are fully methylated. However, we note that we used Bowtie to align the sequenced tags to the human genome and used Sole-Search [11,12] to call peaks. It was possible that this combination of alignment and peak-calling resulted in a loss of the set of highly methylated Kaiso binding sites. To ensure that our method of alignment and peak identification had not biased our results, we reanalyzed the Kaiso ChIP-seq data using LONUT, a new alignment tool which takes into account both unique and non-unique tags (manuscript submitted) and used BELT [29] to call peaks. Also, to eliminate the possibility that we are missing a set of Kaiso peaks that are highly methylated, but are not as robust or not as reproducible as the peaks called using Sole-Search, we relaxed our stringency and called peaks on the merged Kaiso ChIP-seq datasets for each cell type (thus not requiring that each peak be identified in both replicates). We identified many thousands of Kaiso binding sites in GM12878 and K562 cells, most of which were very small and found in only one of the two replicates. We found that most of the Kaiso binding sites identified using this method again had low levels of DNA methylation (Additional file 10). In fact, only 12 of the 8,069 Kaiso peaks identified by LONUT in GM12878 had greater than 50% DNA methylation. Visual inspection of these 12 sites revealed two peaks on the X chromosome (red arrows, Additional file 10A) within the CpG island promoters of the PHF8 and PRDX4 genes (the other 10 sites had low Kaiso peaks and/or were not reproducible between the two biological replicates). A central CGCG motif was found to be 73% methylated in PHF8 and 55% methylated in PRDX4. However, upon further characterization of these promoters, they were discovered to be expressed, and have high levels of active histone modifications surrounding the promoter (Additional file 10B, PHF8 shown). For both promoters, the methylated CGCG motif identified was discovered to be just outside the borders of the Sole-Search-called peaks and therefore was not analyzed in Figure 5. In general, the combination of LONUT and BELT results in the assignment of larger genomic regions to peaks than does the combination of Bowtie and Sole-Search; as a result the new analysis included some new CGCG motifs in the final dataset. However, these CGCG motifs were not highly methylated and the ones that had modest methylation were on the edges of the peak and unlikely to contribute to Kaiso binding.

Binding of transcription factors in the context of chromatin is influenced by many factors. For example, recent ChIP-seq analyses have revealed that not all binding sites contain the ‘expected’ motif that was derived from in vitro binding studies [30,31]. In addition, the epigenetic profile of a promoter region can influence binding, with repressive histone marks and nucleosome occupancy limiting access of transcription factors to their binding sites [32-34]. Taken in this context, we believe that although Kaiso may ‘prefer’ to bind to methylated DNA, this is not an option in the context of chromatin. Under conditions in which the CpGs in a promoter are highly methylated, the entire promoter region is often densely wrapped around nucleosomes and in a repressive heterochromatic state. The absence of a nucleosome-free region prevents access of transcription factors to the promoter region and hence prevents access of Kaiso to its methylated motif. Being denied access to the preferred methylated motif, Kaiso instead binds to the same motif in its unmethylated state. It is interesting to note that this disconnect between in vitro and in vivo binding to a methylated motif has been seen by others. Bartels et al. identified RBP-J as a strong binder of methylated DNA in vitro. However, they cannot find evidence that this factor binds to methylated DNA in vivo [27]. Other zinc finger proteins have been implicated in binding methylated DNA. Quenneville et al. [35] showed that a portion of ZFP57 consisting of two zinc fingers can bind to a methylated TGCCGC motif in vitro and they used bisulfite analysis of ChIP DNA to show that ZFP57 can bind to the methylated allele of three imprinted mouse genes. However, the DNA methylation status of the other 11,000 ZFP57 ChIP-seq peaks was not analyzed and therefore the significance of these three sites in the context of the biological function of ZFP57 is not clear. Spruijt et al. [36] used quantitative mass spectrometry to identify proteins that bind to methylated DNA in vitro, identifying the zinc finger factors KLF2, KLF4, and KLF5 as methyl DNA binding proteins. They report that approximately 18% of the KLF4- binding sites in mouse ES cells show high levels of DNA methylation when using a 100 bp window centered on the peak. However, when the analysis is restricted to the binding motif, the DNA methylation levels dropped significantly. Therefore, it is not clear if KLF4 actually binds to methylated DNA in the context of chromatin.

While we were able to identify a small number of methylated promoters to which Kaiso binds, these genes are enriched for active histone modifications and are expressed. The CpG methylated sites were found to be located away from the center of the Kaiso peak and they do not contain the full TCTCGCGAGA motif. Therefore, it is unclear as to whether these sites are relevant to Kaiso binding or to promoter activity. At this point, we cannot rule out that there are methylated TCTCGCGAGA motifs bound by Kaiso in certain cells under certain growth conditions. As noted above, a previous study has reported that Kaiso binds to a methylated MTA2 promoter region in HeLa cells [10]. Unfortunately, we do not have Kaiso ChIP data for HeLa cells and there is no available DNA methylation data from HeLa cells for the precise region of the MTA2 promoter that corresponds to the reported Kaiso binding site. However, in HeLa cells the MTA2 promoter has marks of open chromatin and is bound by Pol2; furthermore, the MTA2 transcript is expressed in HeLa cells (Additional file 11). Therefore, if Kaiso does bind at the MTA2 promoter in HeLa cells, it does not silence transcription. We have examined the relationship of Kaiso binding and MTA2 expression in six other cell types. We find that Kaiso binds to the MTA2 promoter in K562, A459, and SK-N-SH cells, and in all of these cell lines the MTA2 promoter has acetylated histones. Another study [15] suggested that Kaiso binds to and represses the CDKN2A, HIC1, and MGMT promoters in a methylation-dependent manner in HCT116 cells. However, ChIP-seq data indicates that in HCT116 cells Kaiso does not bind within the genomic regions of these promoters. A recent study [37] claims that Kaiso binds the cyclin D1 CpG island promoter via a ‘KBS motif’, TCCTGCNA, 69 bp upstream of the transcription start site and functions to repress expression of the cyclin D1 gene in HCT116 and MCF7 cells. They show that the methylation of three adjacent CpG dinucleotides helps to stabilize Kaiso’s interaction with sequences containing the cyclin D1 promoter using in vitro assays, and that Kaiso binds the region by ChIP. However, ENCODE ChIP-seq data from HCT116 shows that Kaiso is not enriched at the cyclin D1 promoter and RRBS data shows that the region encompassing the KBS motif 69 bp upstream of the transcription start site is unmethylated in both HCT116 and MCF7. The gene is also bound by Pol2 and acetylated histones, and is expressed in both cell lines. Thus, it is hard to find a clear relationship between Kaiso binding and repression of any gene in any cell line. In fact, by comparison of the expression levels of the genes bound by Kaiso in both GM12878 and K562 versus the expression of the genes whose promoters are bound by Kaiso in a cell type-specific manner, we find a positive correlation between Kaiso- binding and gene expression (Figure 12).

Although many promoters are bound by Kaiso in multiple cell types, we did observe cell type-specific binding. While we do not yet fully understand what specifies cell type-specific binding of Kaiso, it appears that in some cases increased DNA methylation and compact chromatin structure may prevent Kaiso from binding in a particular cell type. Our studies also suggest that interaction with other transcription factors can influence cell type-specific binding of Kaiso. Motif analysis indicated that most cell type-specific Kaiso binding sites did not contain the TCTCGCGAGA motif, suggesting that Kaiso may be recruited to these sites in a manner distinct from direct binding to its motif. To test this hypothesis we performed a motif analysis and found motifs for GATA and CTCF family members in the K562-specific Kaiso peaks and for ETS and Runt family members in the GM12878-specific Kaiso peaks. These results suggested that Kaiso might co-localize with these specific factors in GM12878 or K562 cells. ChIP-seq data from the ENCODE project confirmed the validity of these bioinformatic predictions; we found CTCF, GATA1, and GATA2 to be highly-enriched at K562-unique Kaiso binding sites, and RUNX3 and PU.1 to be highly enriched at GM12878-unique Kaiso binding sites. We note that a previous study has shown that Kaiso can interact with CTCF [24]. Taken together with our genome-wide analyses, we suggest that CTCF may help to recruit Kaiso to certain locations in the genome. Physical interaction studies of Kaiso and GATA factors have not been performed; therefore, further work is necessary to determine the significance of these co-localizations. We note that a previous study suggested that Kaiso can interact with ZNF131 [16]. However, the ZNF131 motif was not identified in any of our unbiased motif analyses. We performed a direct search for the ZNF131 motif allowing up to two mismatches, and found that only 24 of 603 GM-specific peaks that lack a Kaiso motif and only 38 of 1855 K56-specific peaks that lack a Kaiso motif contained a match to the ZNF131 motif. There is no ChIP-seq data for ZNF131, but based on motif analyses, it may not play an important role in recruiting Kaiso to the genome. Our results also indicate that nucleosome occupancy plays a role in dictating Kaiso’s cell type-specific binding between GM12878 and K562 (Figure 11). Specifically, binding sites unique to K562 cells show reduced nucleosome occupancy relative to the same genomic loci in GM12878 cells. However, in GM12878 cells, it appears that the positioning of nucleosomes plays a lesser role in differential Kaiso binding between the two cell types.

## Conclusions

In summary, and in contrast to prediction, all of our analyses suggest that there is a strong positive relationship between the binding of Kaiso, the absence of DNA methylation, the presence of active marks on a promoter, and RNA expression levels. Thus, Kaiso does not appear to play a major role in creating a repressive promoter structure. Rather, our studies suggest that instead of recruiting repressive proteins such as HDACs, Kaiso may recruit positively-acting transcription factors. Future studies are needed to more precisely define the mechanism by which Kaiso contributes to the overall expression level of its target genes. Importantly, our findings establish a clear disconnect between in vitro studies and in vivo studies. Rather than DNA methylation of a small motif, the chromatin landscape, along with the cooperation of Kaiso with other factors, dictates Kaiso binding patterns. We suggest that the fact that Kaiso may prefer a methylated motif is not relevant to in vivo function because the methylated Kaiso motifs are not accessible for binding. A recent study has highlighted the fact that two-thirds of the members of the large family of KRAB domain-containing zinc finger proteins contain a specific argenine-histidine linker sequence between the C2H2 zinc finger domains that may allow recognition of methylated cytosines [38]. Future studies may successfully identify a transcription factor that can bind to a methylated motif in the context of chromatin and contribute to gene silencing.

## Methods

### ChIP-seq peak calling and analysis

Two replicate Bowtie-mapped ChIP-seq datasets were downloaded for GM12878, K562, A549, HepG2, HCT116, and SK-N-SH from the UCSC browser. All Kaiso ChIP-seq experiments were carried out in the Myers Lab using Santa Cruz Biotechnology antibody sc-23871. The antibody has been validated to be specific to Kaiso as required by ENCODE standards [13], using Western blot analysis and mass spectrometry. We note that the antibody validation document shows that the levels of Kaiso in GM12878 and K562 are similar. The Kaiso (ZBTB33) antibody validation document from the ENCODE consortium can be found at http://genome-preview.ucsc.edu/cgi-bin/hgEncodeVocab?term=%22ZBTB33%22. Peaks were called for each replicate in each cell type using Sole-Search [11,12]. The parameters used in the Sole-Search program for all Kaiso datasets were as follows: a permutation of 5, fragment length of 150, alpha value of 0.0010, an FDR of 0.0010, and a peak merge distance of 0. To determine the reproducibility between the two replicates for the Kaiso datasets (and to compare Kaiso peaks with other ChIP-seq datasets), the called peak files for each replicate were compared using the overlap analysis tool in Sole-Search [11,12]. A file containing the genomic locations of all CpG islands were downloaded from the UCSC genome browser. To determine the location of Kaiso peaks (and the location of the Pol2 top-ranked peaks versus the Pol2 peaks excluded from analysis) relative to transcription start sites, the location analysis tool of Sole-Search was used. The epigenetic profiles of the Kaiso binding sites were analyzed using tag density plots that were created using Hypergeometric Optimization of Motif EnRichment software (HOMER, http://biowhat.ucsd.edu/homer/index.html) [22]. All histone modification and transcription factor ChIP-seq datasets used in comparison to Kaiso peaks were downloaded from the UCSC genome browser.

### DNA methylation analysis

To determine the DNA methylation status of the genomic regions corresponding to Kaiso peaks, a BigBed file for GM12878 WGBS data was downloaded from the UCSC genome browser and was converted to BED format using the BigBedtoBed script available at http://hgdownload.soe.ucsc.edu/admin/exe/macOSX.i386/. HOMER [22] was then used to plot the percent methylation across Kaiso peak regions using the annotatePeaks.pl script and the ‘-ratio’ option, comparing peak files to tag directories created using the ‘-mCpGbed’ option. The resulting output files were used to create histograms plotting the average percent methylation in 500bp regions surrounding the center of Kaiso peaks using a bin size of 10bp. To identify an enriched motif within Kaiso-binding sites, a motif analysis was performed using HOMER to identify strings of nucleotide sequences that are enriched within Kaiso peaks. The findMotifsGenome.pl command was run in HOMER using hg19 as the reference genome. To limit the motif analysis to regions pulled down by ChIP, the analysis was limited to sequences contained within each peak using the ‘-size given’ option. For some analyses, we centered Kaiso peaks on the methylated motif using the annotatePeaks.pl script and the -center option in HOMER. To determine the methylation status of individual CGCG motifs contained with peaks, the genomic coordinates of each motif was compared to WGBS and/or RRBS data, and a beta value was assigned to each CpG within the motif. For GM12878 methylation analyses, WGBS and RRBS data was combined to achieve higher genomic coverage; for K562 analyses, only RRBS data was available. Only bases with a minimum 3× sequencing coverage were used in methylation analyses.

### RNA-seq analysis

The RefSeq gene annotation files for RNA-seq data in GM12878 and K562 were downloaded from the UCSC genome browser (http://hgdownload.cse.ucsc.edu/goldenPath/hg19/encodeDCC/wgEncodeCaltechRnaSeq/). A union file was created by combining all transcripts for individual genes with multiple transcripts. For both cell types, all replicates were combined for analysis. All reads were aligned to the hg19 reference genome using the bowtie aligner, and those uniquely mapped were used to calculate the expression level as reads per kilobase per million reads (RPKM), using the following formula:

$$\mathrm{RPKM}=n/\mathrm{NL}\times 1.0\times 10^{8}$$

where n is the number of mapped reads localized within exons, N is the total number of uniquely mapped reads in the experiment, and L is the length of gene body summing from all union exons in base pairs. We note that Kaiso had an RPKM value of 0.004081966 in GM12878 and 0.004755255 in K562, and ranked within the top 40% of expressed genes in both cell types. To verify the difference among groups of peaks (K562-unique, common, GM12878-unique), the Mann-Whitney rank test was applied.

## Abbreviations

Bp: Base pairs; ChIP: Chromatin immunoprecipitation; EBV: Epstein-Barr virus; HDAC: Histone deacetylase; HOMER: Hypergeometric Optimization of Motif EnRichment; MNase: Micrococcal nuclease; RPKM: Reads per kilobase per million reads; RRBS: Reduced representation bisulfite sequencing; WGBS: Whole genome bisulfite sequencing.

## Competing interests

The authors declare that they have no competing interests.

## Authors’ contributions

AB carried out the analyses and interpretation of ChIP-seq and MNase-seq data and drafted the manuscript. LY performed analyses of the DNA methylation, YW performed LONUT alignments and BELT peak calling, ZY performed the analysis of RNA-seq data, VXJ coordinated LONUT and BELT ChIP-seq data analysis and RNA-seq data analysis, and PJF conceived of the study, participated in its design and coordination, and helped to draft the manuscript. All authors have read and approved the final manuscript.

## Supplementary Material

Additional file 1 Chromosomal locations and peak scores for Kaiso peaks sets in all cell lines. Click here for file

Additional file 2 **Peak selection for Pol2 in GM12878 cells. Shown are the peaks called using Sole-Search for the merged replicate datasets of Pol2 in GM12878 (panel A).** The parameters used in the Sole-Search peak calling were as follows: permutation of 5, fragment length of 150, alpha value of 0.0010, an FDR of 0.0010, and a peak merge distance of 0. The top 20% of the called peaks were used (tag height > 50). The arrow indicates the position at which the peak list was truncated. Panel B shows the location analysis for Pol2 peaks that were used in our analysis (red) and those that were discarded (blue). Click here for file

Additional file 3 HOMER Annotation of Kaiso peak files in GM12878 and K562, including locations of Kaiso motifs. Click here for file

Additional file 4 Chromosomal location and percent methylation of CGCG motifs within GM12878 and K562 Kaiso peaks. Click here for file

Additional file 5 **Motif analysis of subsets of K562 Kaiso peaks identified in highly amplified genomic regions.** As shown, the Kaiso motif is not highly represented in the removed peaks. The adjusted high-confidence peak set is used for analyses in this paper. However, the genomic coordinates of the retained and removed peaks are provided in Additional file 6. Click here for file

Additional file 6 Peak files used in the removal of amplified regions in K562 cells. Click here for file

Additional file 7 **Methylation of Kaiso peaks in K562. Snapshot of the region on chromosome 6 containing the highest methylated CGCG motif identified within Kaiso K562 high-confidence peaks.** The ChIP-seq track for Kaiso is shown in black, with called peaks represented by black bars below the track. ChIP-seq tracks for Pol2 and histone modifications are shown in blue. The inset shows a zoom in of the region bound by Kaiso containing a methylated CGCG motif. Red bars in the RRBS track represent methylated cytosines. Click here for file

Additional file 8 **Motif analysis of cell type-specific peaks lacking a Kaiso motif.** (A) K562 Kaiso peaks that lack a Kaiso motif were searched for known motifs for other TFs using Homer. (B) GM12878-unique distal Kaiso peaks that lack a Kaiso motif were searched for known motifs for other TFs using Homer. Click here for file

Additional file 9 **Plot of DNA methylation versus peak rank.** The methylation status of each CGCG motif within Kaiso peaks was calculated using WGBS and RRBS data (for GM12878, left panel) and RRBS (for K562, right panel), and plotted relative to the score of the peak containing the motif. The left panel shows high-confidence Kaiso peaks in GM12878 and the right panel shows high- confidence Kaiso peaks in K562. Click here for file

Additional file 10 **Methylation of Kaiso peaks identified by LONUT.** (A) Comparison of methylation levels within peaks called by different programs. Peaks were called by BELT using non-uniquely mapped tags (left) and uniquely-mapped tags (middle) that were mapped using LONUT. These peaks were compared to Sole-Search-called peaks (right) for methylation of CGCG motifs. Red arrows indicate CGCG motifs identified in the promoters of the PHF8 and PRDX4 genes. (B) Genome browser snapshot of the region surrounding the PHF8 gene, where a CGCG motif is 73% methylated in GM12878 cells. ChIP-seq tracks for Pol2 and histone modifications are shown in red and the Kaiso ChIP-seq track is shown in black. Click here for file

Additional file 11 **MTA2 promoter showing no DNA methylation and active promoter marks in HeLa cells.** Genome browser snapshot showing the MTA2 gene. The region analyzed for Kaiso-binding and DNA methylation in Yoon et al. is represented by the black box under the red arrow. RRBS tracks for several cell types are shown in green, red, and yellow, but is absent in all cell lines for the region in question. ChIP-seq density tracks for Pol2 and histone modifications in HeLa cells are shown in grey-scale. Click here for file
