# Supplementary figures and images for: ZBTB33 binds unmethylated regions of the genome associated with actively expressed genes
Source: Epigenetics Chromatin. 2013 May 21;6:13. doi: 10.1186/1756-8935-6-13 (PMC3663758; doi:10.1186/1756-8935-6-13)

## Blattler Additional File 2

A

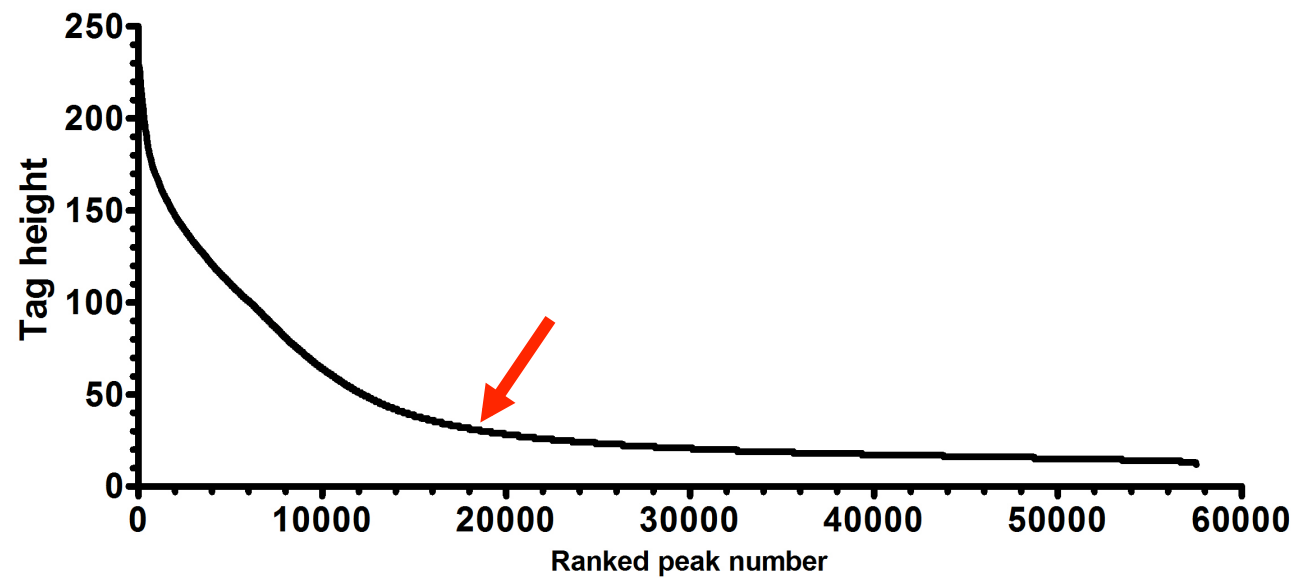

B

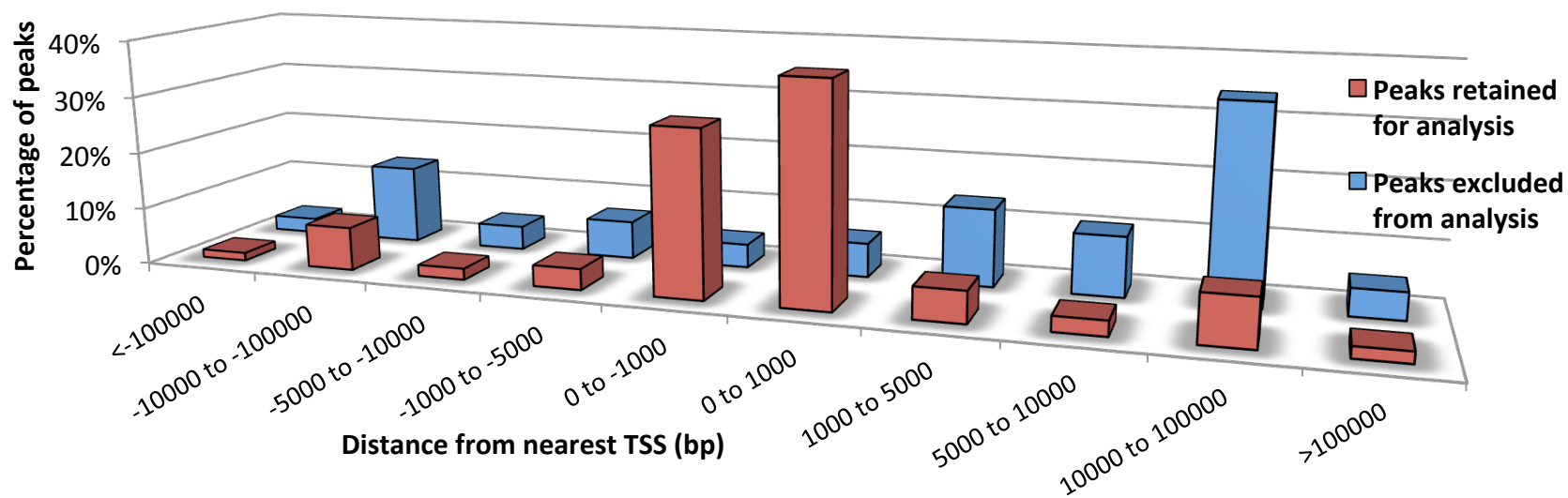

Supplement: Additional file 2 — Peak selection for Pol2 in GM12878 cells. Shown are the peaks called using Sole-Search for the merged replicate datasets of Pol2 in GM12878 (panel A). The parameters used in the Sole-Search peak calling were as follows: permutation of 5, fragment length of 150, alpha value of 0.0010, an FDR of 0.0010, and a peak merge distance of 0. The top 20% of the called peaks were used (tag height > 50). The arrow indicates the position at which the peak list was truncated. Panel B shows the location analysis for Pol2 peaks that were used in our analysis (red) and those that were discarded (blue). [file 1756-8935-6-13-S2.pdf]

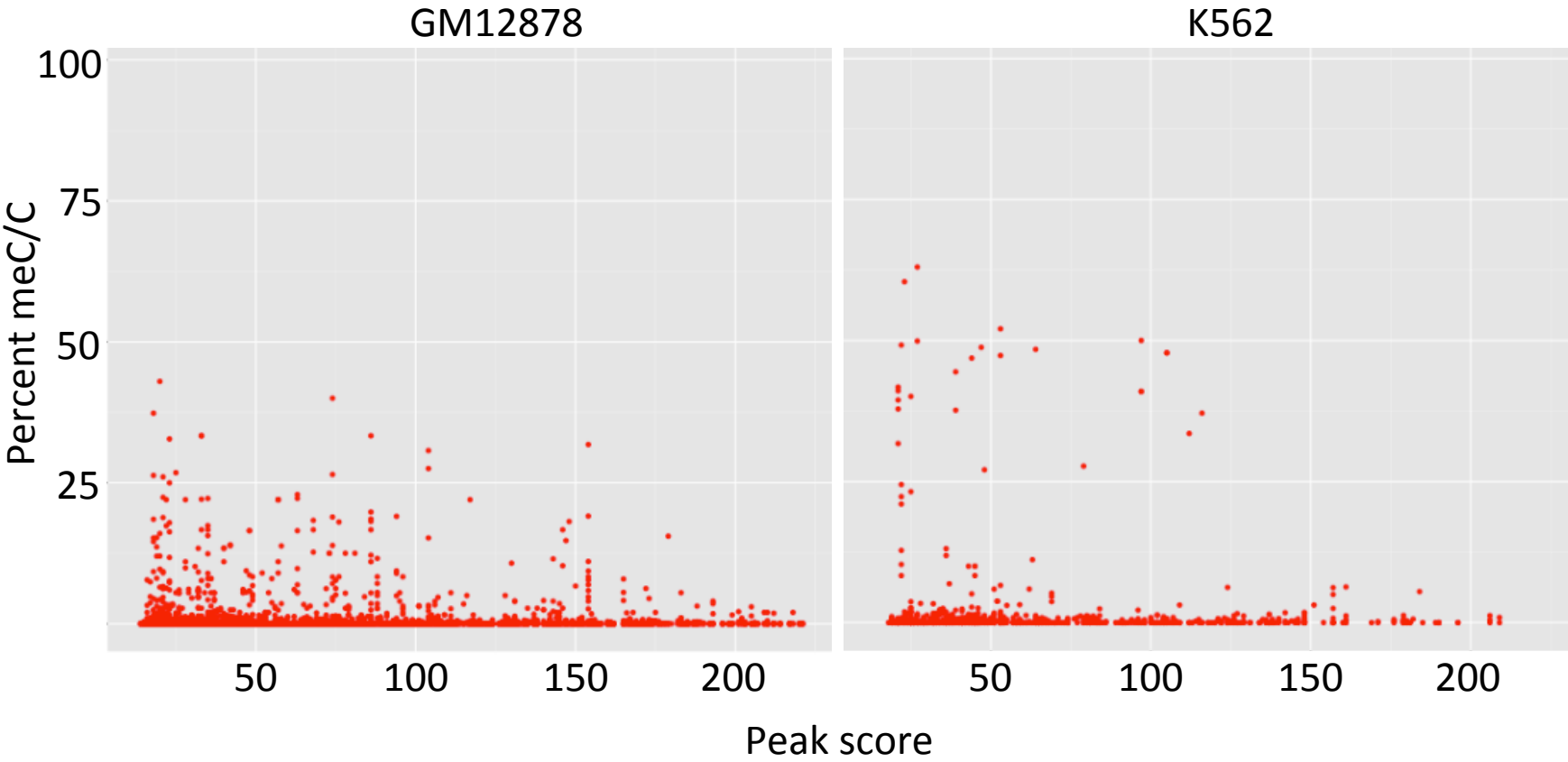

Supplement: Additional file 9 — Plot of DNA methylation versus peak rank. The methylation status of each CGCG motif within Kaiso peaks was calculated using WGBS and RRBS data (for GM12878, left panel) and RRBS (for K562, right panel), and plotted relative to the score of the peak containing the motif. The left panel shows high-confidence Kaiso peaks in GM12878 and the right panel shows high- confidence Kaiso peaks in K562. [file 1756-8935-6-13-S9.pdf]

# Blattler Additional File 10

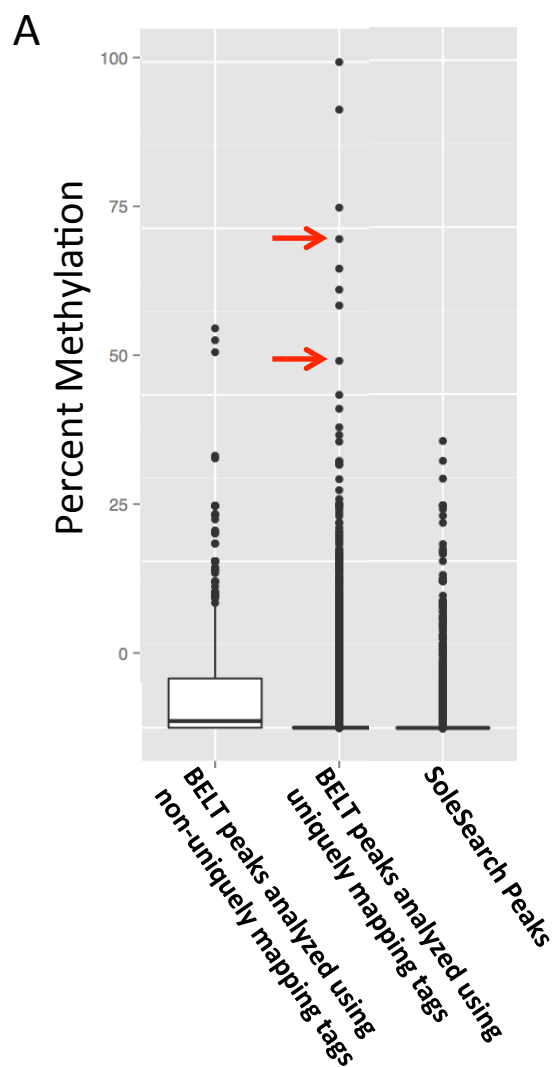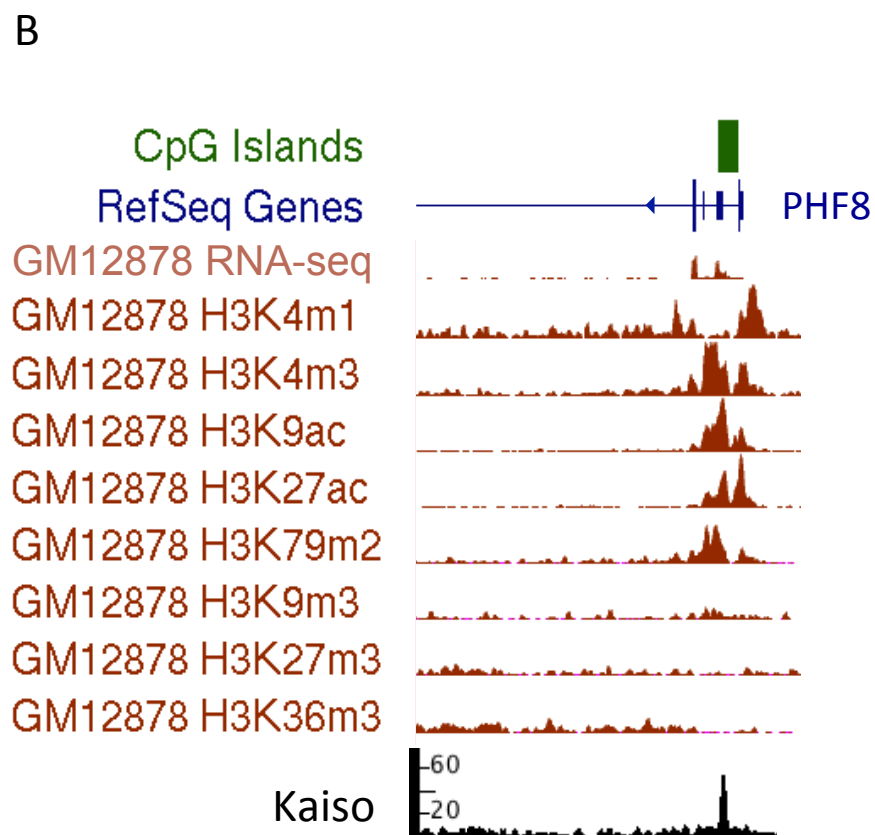

Supplement: Additional file 10 — Methylation of Kaiso peaks identified by LONUT. (A) Comparison of methylation levels within peaks called by different programs. Peaks were called by BELT using non-uniquely mapped tags (left) and uniquely-mapped tags (middle) that were mapped using LONUT. These peaks were compared to Sole-Search-called peaks (right) for methylation of CGCG motifs. Red arrows indicate CGCG motifs identified in the promoters of the PHF8 and PRDX4 genes. (B) Genome browser snapshot of the region surrounding the PHF8 gene, where a CGCG motif is 73% methylated in GM12878 cells. ChIP-seq tracks for Pol2 and histone modifications are shown in red and the Kaiso ChIP-seq track is shown in black. [file 1756-8935-6-13-S10.pdf]

Blattler Additional File 11

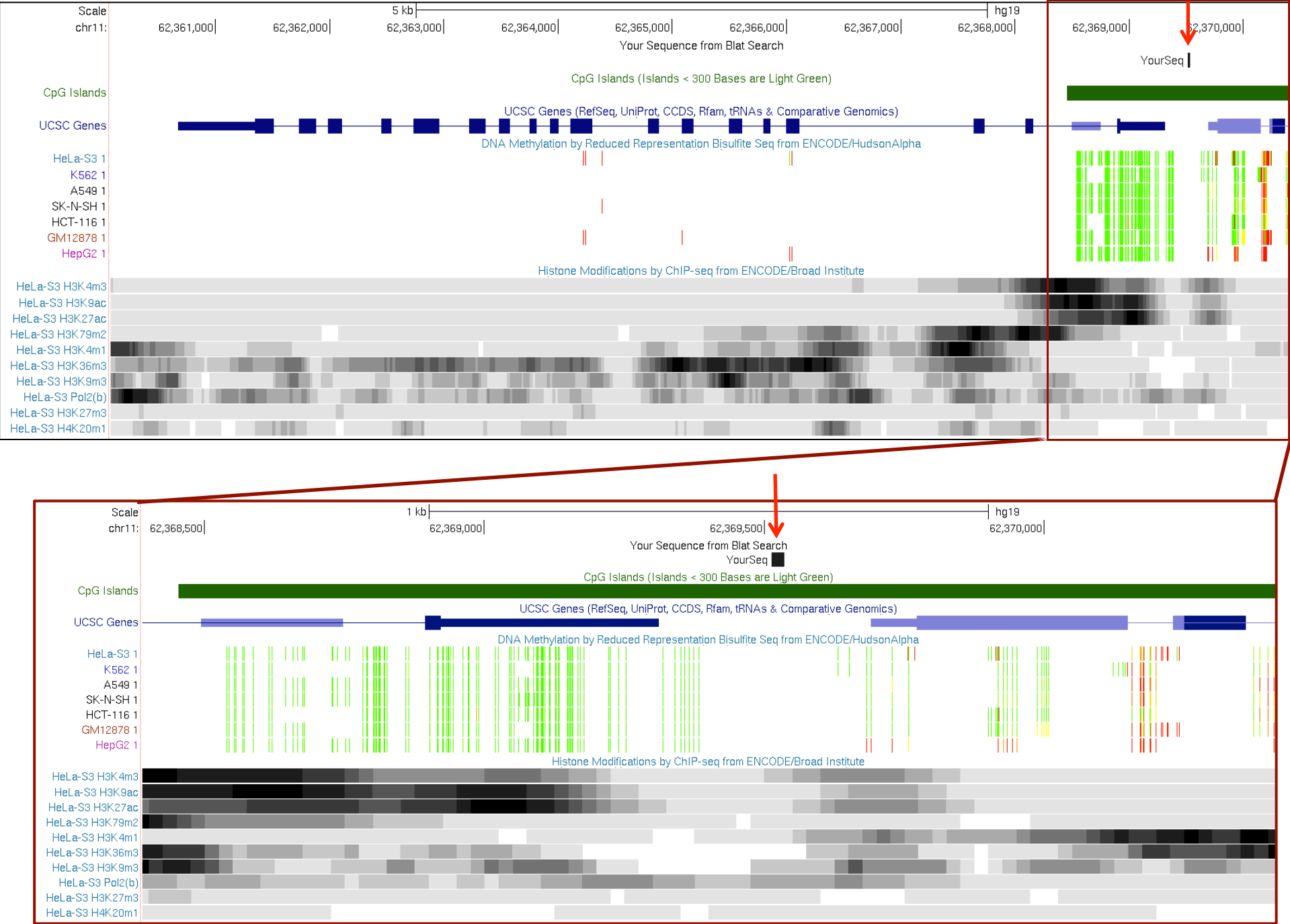

Supplement: Additional file 11 — MTA2 promoter showing no DNA methylation and active promoter marks in HeLa cells. Genome browser snapshot showing the MTA2 gene. The region analyzed for Kaiso-binding and DNA methylation in Yoon et al. is represented by the black box under the red arrow. RRBS tracks for several cell types are shown in green, red, and yellow, but is absent in all cell lines for the region in question. ChIP-seq density tracks for Pol2 and histone modifications in HeLa cells are shown in grey-scale. [file 1756-8935-6-13-S11.pdf]
