# Supplementary material for: ZBTB33 binds unmethylated regions of the genome associated with actively expressed genes
Source: Epigenetics Chromatin. 2013 May 21;6:13. doi: 10.1186/1756-8935-6-13 (PMC3663758; doi:10.1186/1756-8935-6-13)
Supplement: Additional file 5 — Motif analysis of subsets of K562 Kaiso peaks identified in highly amplified genomic regions. As shown, the Kaiso motif is not highly represented in the removed peaks. The adjusted high-confidence peak set is used for analyses in this paper. However, the genomic coordinates of the retained and removed peaks are provided in Additional file 6. [file 1756-8935-6-13-S5.pdf]

Blattler Additional File 5

Percentage of peaks containing each motif		
	TCTCGCGAGA	CGCG
K562 high-confidence peaks (8,296 peaks)	12%	25%
K562 Adjusted high-confidence peaks - without amplified regions (3,082 peaks)	29%	47%
Removed peaks- from the K562 amplified regions (5,214 peaks)	3%	13%
