# Supplementary material for: ZBTB33 binds unmethylated regions of the genome associated with actively expressed genes
Source: Epigenetics Chromatin. 2013 May 21;6:13. doi: 10.1186/1756-8935-6-13 (PMC3663758; doi:10.1186/1756-8935-6-13)
Supplement: Additional file 7 — Methylation of Kaiso peaks in K562. Snapshot of the region on chromosome 6 containing the highest methylated CGCG motif identified within Kaiso K562 high-confidence peaks. The ChIP-seq track for Kaiso is shown in black, with called peaks represented by black bars below the track. ChIP-seq tracks for Pol2 and histone modifications are shown in blue. The inset shows a zoom in of the region bound by Kaiso containing a methylated CGCG motif. Red bars in the RRBS track represent methylated cytosines. [file 1756-8935-6-13-S7.pdf]

Blattler Additional File 7

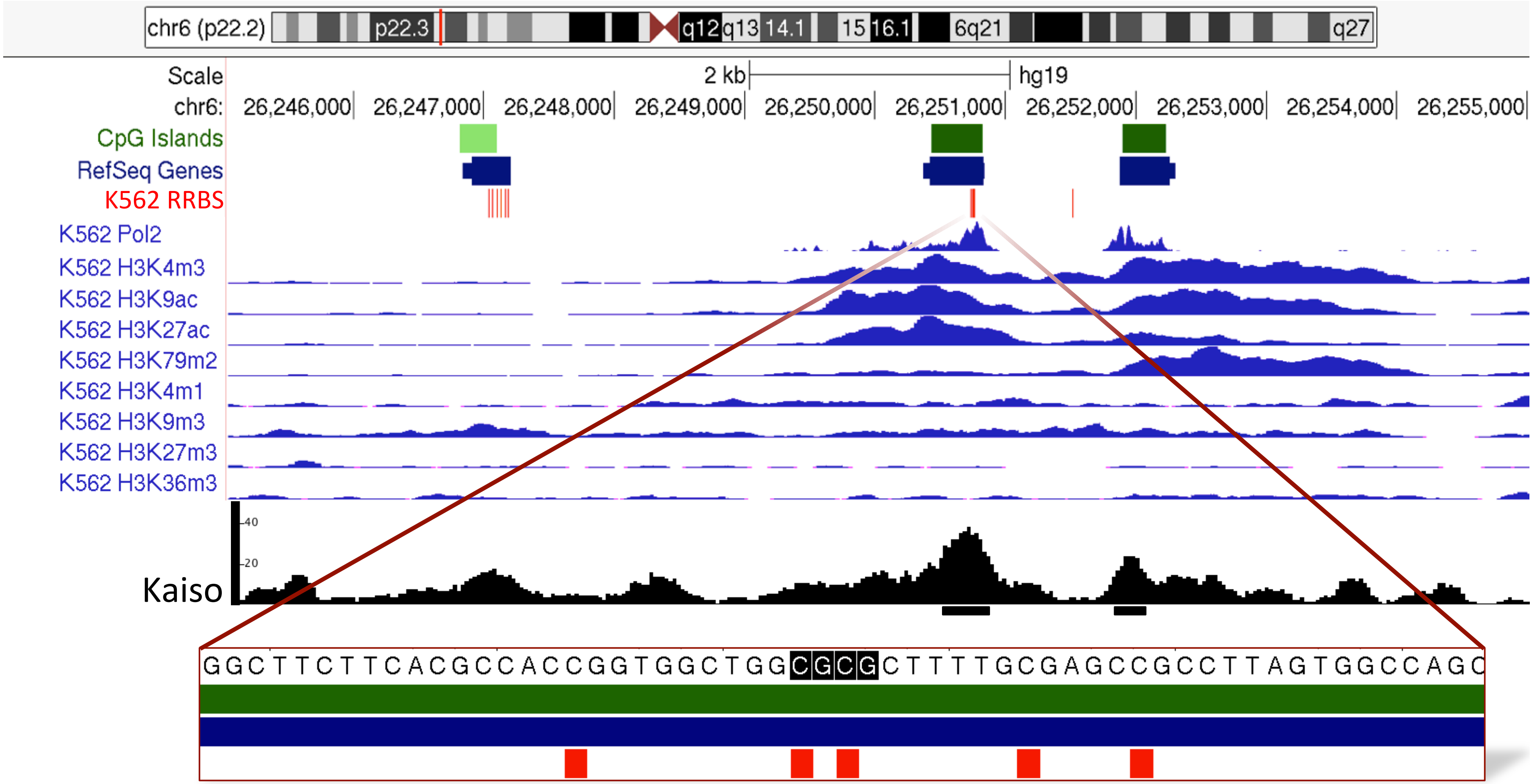

The RRBS track uses an 11-color gradient to represent the methylation status of each cytosine analyzed. Red ( ) is 100% methylated, yellow ( ) is 50% methylated, and green ( ) is 0% methylated.
