# Supplementary material for: ZBTB33 binds unmethylated regions of the genome associated with actively expressed genes
Source: Epigenetics Chromatin. 2013 May 21;6:13. doi: 10.1186/1756-8935-6-13 (PMC3663758; doi:10.1186/1756-8935-6-13)
Supplement: Additional file 8 — Motif analysis of cell type-specific peaks lacking a Kaiso motif. (A) K562 Kaiso peaks that lack a Kaiso motif were searched for known motifs for other TFs using Homer. (B) GM12878-unique distal Kaiso peaks that lack a Kaiso motif were searched for known motifs for other TFs using Homer. [file 1756-8935-6-13-S8.pdf]

Blattler Additional File 8A

Rank	Motif	P-value	
1	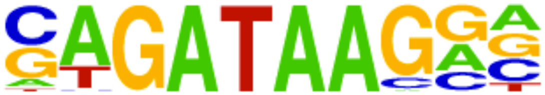	1e-41	GATA1
2	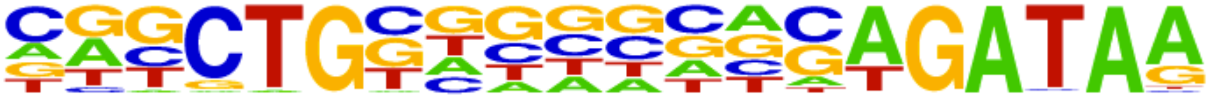	1e-35	GATA
3	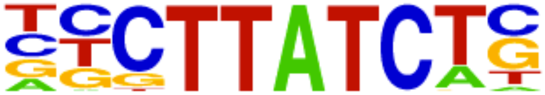	1e-35	GATA2
4	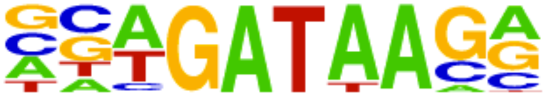	1e-26	GATA4
5	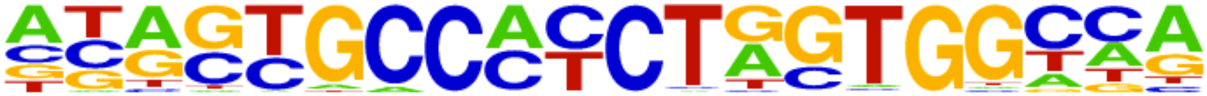	1e-15	CTCF
6	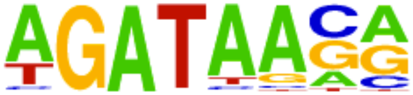	1e-11	GATA3
7	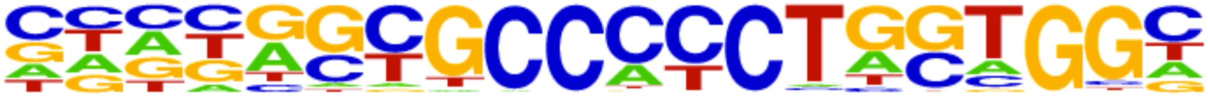	1e-10	CTCFL
8	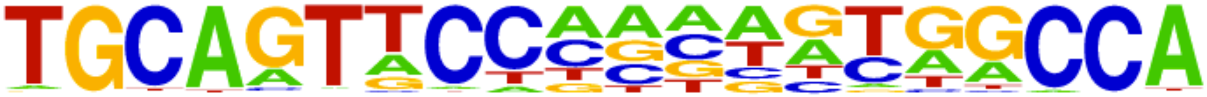	1e-5	CTCF

Blattler Additional File 8B

Rank	Motif	P-value	
1	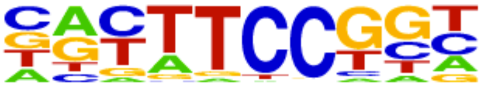	1e-15	**FLi1 (ETS)**
2	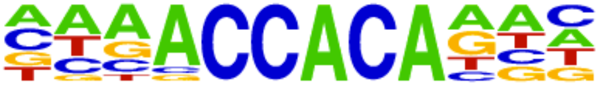	1e-12	**RUNX2 (Runt)**
3	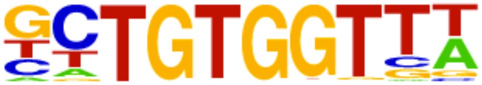	1e-12	**RUNX (Runt)**
4	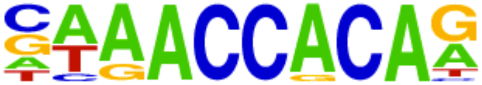	1e-11	**RUNX1 (Runt)**
5	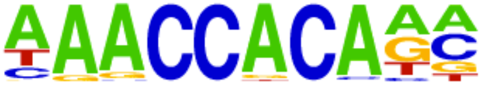	1e-11	**RUNX1 (Runt)**
6	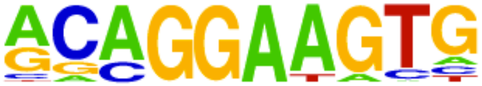	1e-10	**ETS1 (ETS)**
7	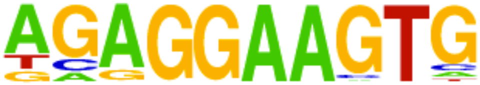	1e-8	**PU.1 (ETS)**
8	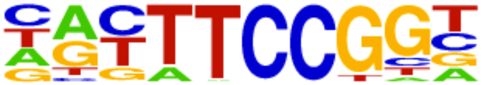	1e-8	**ELK4 (ETS)**
9	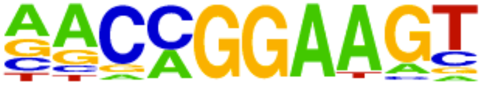	1e-8	**ETV1 (ETS)**
